# Prenatal and Postnatal Epigenetic Programming: Implications for GI, Immune, and Neuronal Function in Autism

**DOI:** 10.1155/2012/190930

**Published:** 2012-06-19

**Authors:** Mostafa I. Waly, Mady Hornig, Malav Trivedi, Nathaniel Hodgson, Radhika Kini, Akio Ohta, Richard Deth

**Affiliations:** ^1^Department of Food Science and Nutrition, Sultan Qaboos University, Alkoudh 123, Muscat, Oman; ^2^Department of Epidemiology, Columbia University, New York, NY 10032, USA; ^3^Department of Pharmaceutical Sciences, Northeastern University, Boston, MA 02115, USA

## Abstract

Although autism is first and foremost a disorder of the central nervous system, comorbid dysfunction of the gastrointestinal (GI) and immune systems is common, suggesting that all three systems may be affected by common molecular mechanisms. Substantial systemic deficits in the antioxidant glutathione and its precursor, cysteine, have been documented in autism in association with oxidative stress and impaired methylation. DNA and histone methylation provide epigenetic regulation of gene expression during prenatal and postnatal development. Prenatal epigenetic programming (PrEP) can be affected by the maternal metabolic and nutritional environment, whereas postnatal epigenetic programming (PEP) importantly depends upon nutritional support provided through the GI tract. Cysteine absorption from the GI tract is a crucial determinant of antioxidant capacity, and systemic deficits of glutathione and cysteine in autism are likely to reflect impaired cysteine absorption. Excitatory amino acid transporter 3 (EAAT3) provides cysteine uptake for GI epithelial, neuronal, and immune cells, and its activity is decreased during oxidative stress. Based upon these observations, we propose that neurodevelopmental, GI, and immune aspects of autism each reflect manifestations of inadequate antioxidant capacity, secondary to impaired cysteine uptake by the GI tract. Genetic and environmental factors that adversely affect antioxidant capacity can disrupt PrEP and/or PEP, increasing vulnerability to autism.

## 1. Introduction

Neurological and behavioral symptoms implicate abnormal brain development as a core pathophysiological feature of autism, but increasing evidence also indicates immune [1–8] and gastrointestinal (GI) abnormalities in a significant subset [1, 8–12]. This triad of dysfunctional systems provides not only a more complete description of autism and autism spectrum disorders (ASDs), but also an important opportunity to consider the mechanisms which could result in the shared involvement of these three particular systems, especially in the context of development. Recent elucidation of the central role of epigenetic regulation of gene expression in development presents a molecular framework within which prenatal and postnatal maturation of neuronal, immune, and GI systems can be viewed. 

As all cells possess the same DNA, their differentiation into various cell types reflects stable suppression of some genes and activation of others, accomplished in large part by epigenetic regulation. Such regulation involves reversible modifications of both DNA nucleotides and histone proteins [13]. These modifications or epigenetic marks favor or disfavor formation of nucleosomes, DNA/histone complexes that maintain genes in a compacted, inactive state (heterochromatin). Methylation of DNA is the most fundamental epigenetic mark, enabling binding of proteins containing methylDNA binding domains, such as methyl CpG binding protein 2 (MeCP2). Proteins with methylDNA binding domains recruit other proteins, including those capable of modifying histones at their tail regions [14]. Multiple sites and forms of histone modification (e.g., methylation, acetylation, and phosphorylation) make this a highly complex mode of regulation, affected by diverse signaling pathways that adjust gene expression to local cellular metabolic conditions [15]. Whereas certain epigenetic marks are to some degree reversible, others are not readily reversed and, once in place, these may be sustained for an entire lifespan and/or be transmitted across generations through germline modifications [16]. Accordingly, epigenetic marking or programming early in development has the potential to exert long-lasting effects [17]. 

There is considerable evidence that epigenetic programming is highly sensitive to changes in the cellular environment, as broadly defined [18]. Indeed, it appears that epigenetic regulation is a widely utilized adaptive mechanism to allow cells to maintain a favorable metabolic status under different conditions, including differential exposure to physiological substances (e.g., hormones, neurotransmitters, or growth-regulation factors), xenobiotics (e.g., pollutants, toxic chemicals), or even infectious agents (e.g., bacteria or viruses, fungi or parasites) [19]. Epigenetic regulation may facilitate adaptation to changes in the cellular environment through stable alterations of cellular phenotype, potentially resulting in progressive differentiation and maturation during fetal and possibly postnatal development [20]. 

During prenatal development, nutritional support to the fetus is provided via the transplacental circulation as a function of the available maternal nutriture, with metabolic consequences for both the mother and developing child. During postnatal development, nutritional support is provided by oral food intake into the GI tract: breast-milk- or cow-milk-based formula at first, followed by introduction of other foods, usually in a controlled, gradual manner, but the provision of adequate nutritional resources to support further development of the newborn is not assured. Thus, the prenatal/postnatal transition is a critical juncture in metabolic adaptation. This transition is particularly linked to aerobic metabolism, as the delivery of oxygen via newborn lungs and its ultimate rate of utilization must be balanced by the available antioxidant capacity of body tissues. Epigenetic regulation offers an opportunity for adaptation during this metabolic transition, allowing the level of ongoing aerobic metabolism in each organ system, tissue, and cell type to be maintained in homeostatic equilibrium with antioxidant metabolism. 

We use the term postnatal epigenetic programming (PEP) to describe the ongoing adaptive changes in gene expression occurring in response to the transition from fetal to postnatal metabolism, as distinct from prenatal epigenetic programming (PrEP), at term that recognizes the dynamic occurrence of similar changes which occur *in utero*. While it is clear that autism can result from a variety of genetic and environmental factors, by focusing on factors affecting antioxidant and methylation status in the developing GI tract, immune system, and brain, we hope to illuminate the pathological mechanisms underlying ASDs and other related disorders. 

## 2. Materials and Methods

### 2.1. Purification of Regulatory T Cells

To purify CD4^+^ CD25^+^ regulatory T cells, spleen cells and lymph node cells from C57BL/6 mice were combined as a cell source. The cells were labeled with FITC-conjugated anti-CD24 and anti-CD8 mAbs and with anti-FITC microbeads. After the removal of CD8^+^ T cells and CD24-expressing B cells using an AutoMACS separator (Miltenyi Biotec, Auburn, CA), regulatory T cells were purified by positive selection of CD25^+^ cells using PE-conjugated anti-CD25 mAb and anti-PE microbeads. All antibodies except for anti-CD25 mAb were from BD Biosciences (San Jose, CA). Other materials were from Miltenyi Biotec. Purity of CD4^+^ CD25^+^ cells was higher than 95 %.

### 2.2. RNA Isolation

RNA was isolated using the RNAqueous-4PCR kit (Ambion, Naugatuck, CT), then treated with DNase to eliminate contaminating genomic DNA, and quantified using an ND-1000 NanoDrop spectrophotometer.

### 2.3. cDNA Synthesis

cDNA synthesis was performed using the First strand cDNA synthesis (Roche, Nutley, NJ). 1 *μ*g RNA was used as template, along with 1 mM dNTP mix, 60 uM random hexamer primers and dH_2_0 to make a final volume of 13 *μ*L. Samples were denatured at 65°C for 5 minutes and then placed on ice. Transcriptor RT (20 units/*μ*L), Protector RNase inhibitor (40 U/*μ*L), 5x Transcriptor Reverse Transcriptase Reaction Buffer, and dH_2_0 in a final volume of 7 *μ*L were used to bring the final volume to 20 *μ*L. This was followed by incubation at 25°C for 10 min followed by 30 min incubation at 55°C. The reverse transcriptase enzyme was inhibited by incubating at 85°C for 5 min and then cooling on ice. 

### 2.4. Primers

Primers were designed using the Invitrogen OligoPerfect Designer to have approximately 50–60% GC content, an annealing temperature of 60°C, and a length of 20 bases. Glyceraldehyde phosphate dehydrogenase (GAPDH) expression was used for normalization. Primers for EAAT3 (designated EAAC1 in mice) and GAPDH were EAAT3-Forward: 5′-TTACAGCCACCGCTGCCAGC-3′; EAAT3-Reverse: 5′-GGCAGCCCCACAGCACTCAG-3′; GAPDH-Forward: 5′-TCTCCACACCTATGGTGCAA-3′; GAPDH-Reverse: 5′-CAAGAAACAGGGGAGCTGAG-3′.

### 2.5. qRT-PCR Analysis

qRT-PCR was performed on duplicate samples using the LightCycler 480 from Roche. 5 *μ*L of cDNA was used for qRTPCR, along with 10 *μ*M sense and antisense primers, 10 *μ*L SYBR Green I Master from Roche, and dH20 in a final volume of 20 *μ*L. The following thermal parameters were used: incubation for 5 min at 95°C, followed by 45 cycles of 95°C for 10 sec, 60°C for 20 sec and 72°C for 30 sec, and lastly a single cycle of 95°C for 5 sec, 1 min at 65°C and 97°C for the melting curve. No template controls were run on each plate and dissociation curves were generated to determine any nonspecific products. Data was normalized to GAPDH, and the ΔCt second derivative method was used for analysis of EAAT3 expression.

### 2.6. MS Assay

Brain cortex samples were obtained from thimerosal-treated or untreated C57BL/6 and SJL/J mice, as per the previously published protocol [21]. MS activity in mouse cortex was determined by measuring incorporation of radiolabel from [methyl-^14^C]methyltetrahydrofolate into methionine, as previously described [22]. Enzyme assays were performed under anaerobic conditions by bubbling nitrogen gas through stoppered vials for 1 hour at 37°C and terminated by heating at 98°C for 2 min after which samples were cooled on ice. Radiolabeled methionine was separated from unreacted radiolabeled methylfolate by passing the reaction mixture through an anion exchange column. The column was washed with 2 mL of water and the aqueous samples were collected and counted. Reported values are normalized to protein content and corrected for the counts observed in control assays in which sample enzyme was omitted. 

### 2.7. GSH Measurement

A 100 *μ*L aliquot of cortex supernatant was incubated with 2 *μ*L of monochlorobimane (25 mmol/L) and 2 *μ*L of glutathione-S-transferase reagent was added, as provided by a commercial kit (Biovision, Mountain View, CA). After a 30 min incubation at 37°C, fluorescence was read at 380 nm excitation and 460 nm emission. GSH content was determined by comparison with values from a standard curve using freshly prepared GSH. 

### 2.8. Statistical Methods

 Statistical analysis was carried out using Graph Pad Prism version 5.01. Data was expressed as mean ± standard error of the mean (SEM). Student's *t*-test was conducted to determine the differences between individual groups.

## 3. Results and Discussion

### 3.1. Antioxidant Capacity and Aerobic Metabolism

Aerobic metabolism is unavoidably accompanied by the risk of oxidative damage, most notably caused by reactive oxygen species (ROS) generated as by-products of mitochondrial oxidative phosphorylation and ATP production. Increased metabolic activity requires higher rates of ATP production and increased ROS formation. Maintenance of redox equilibrium requires inactivation of ROS before these by-products can oxidize cellular components, as well as repair of oxidized biomolecules (proteins, lipids, DNA, and RNA). Catalase, superoxide dismutase and the selenoprotein glutathione peroxidase inactivate ROS, and a wide array of redox-active proteins and enzymes participate in the repair of oxidized molecules and the maintenance of thiols in their reduced state, with glucose-derived NADPH serving as the source of reducing electrons [23, 24].

As elegantly reviewed by Schafer and Buettner [25], the equilibrium between reduced and oxidized forms of glutathione (GSH and GSSG, resp.) is the primary determinant of intracellular redox status. The relatively high concentration of GSH within the cell imposes a pervasive influence on essentially all aspects of metabolism. Although all cell types and tissues rely upon the same core antioxidant mechanisms, variations on this central theme are recognized. For example, the intracellular level of GSH in liver hepatocytes is close to 10 mM [25], whereas in neurons it is 0.2 mM, some 50-fold lower [26].

As the rate of ROS formation must be quantitatively balanced by the available antioxidant capacity, a multitude of mechanisms have evolved to restrict aerobic metabolism when antioxidant status is low and vice versa. One prominent example involves glutathionylation of cysteine residues in complex I of the mitochondrial electron transport chain, which limits the flow of electrons, thereby restricting downstream ROS formation [27]. In glutathionylation, GSSG, the oxidized form of glutathione, reacts with a reduced cysteine, resulting in GSH release and modification of the protein in proportion to the prevailing redox state [28]. This regulatory mechanism not only controls ROS formation but also provides an auxiliary pathway for glutathione reductase activity and production of GSH. Additional intracellular control over the balance between ROS and antioxidant reservoirs is achieved by the capacity for reversal of the glutathionylation of complex I by the action of glutaredoxin 2, in conjunction with the selenoprotein thioredoxin reductase, using NADPH-derived electrons [27]. 

More than ten studies have reported significantly lower plasma levels of GSH in autism, accompanied by lower levels of cysteine [29–39]. Among these studies, the average decrease in GSH was 37%, representing a substantial reduction in antioxidant capacity. Several studies also reported a significant decrease in the GSH to GSSG ratio [8–20, 22–36], indicative of oxidative stress, consistent with other reports of increased biomarkers of oxidative stress [29, 32, 40]. One consequence of persistent oxidative stress, an increase in glutathionylation of complex I of the mitochondrial electron transport chain, provides a potential explanation for the mitochondrial dysfunction frequently reported in autism [7]. Genetic factors affecting mitochondrial function may interact with oxidative stress. For example, SLC25A12, the gene encoding the Ca^2+^-dependent aspartate-glutamate carrier isoform 1 (AGC1), has been identified as a putative autism susceptibility gene [41]. AGC1 facilitates the supply of NADH for oxidative phosphorylation, and elevated Ca^2+^ levels will, therefore, increase ROS formation via increased AGC1 activity. Higher Ca^2+^-dependent AGC1 transport rates were demonstrated in autistic subjects [41], and it has been proposed that dyregulated Ca^2+^ levels may be a broadly influential factor in causing autism [42].

### 3.2. Intestinal Absorption of Cysteine

**Figure 1:**
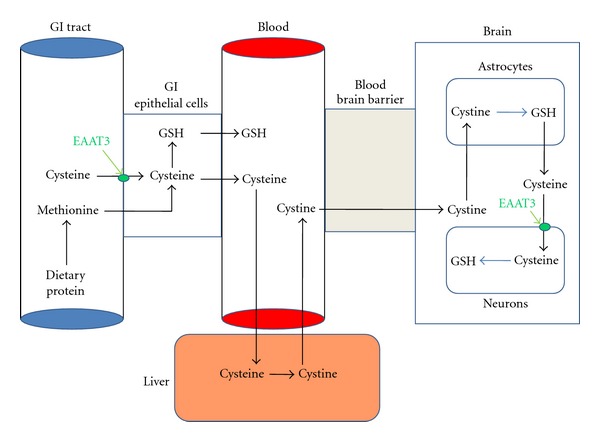
Absorption and systemic distribution of dietary cysteine and methionine. GI epithelial cells take up cysteine and methionine, with EAAT3 being particularly important for cysteine uptake in the distal ileum. A portion of methionine is converted to cysteine via the methionine cycle and transsulfuration of homocysteine. Absorbed cysteine is taken up by the liver, which releases oxidized cystine. Cystine, but not cysteine, is able to traverse the blood brain barrier and is taken up by astroctyes, which convert it to GSH. Cysteine from astrocyte-derived GSH is available for EAAT3-mediated neuronal uptake, supporting neuronal GSH synthesis.

Starting from birth, the systemic availability of cysteine to support GSH synthesis is dependent upon absorption of the food-derived sulfur-containing amino acids cysteine and methionine from the GI tract (Figure 1). Adequate GI uptake of selenium is also critical for maintaining GSH in its reduced state. Intestinal epithelial cells express different transporters on their luminal brush border surface that are critical for uptake of sulfur and selenium-containing amino acids. These amino acids are then subject to intracellular metabolism before being ultimately released on the contralateral surface, either in their free form or incorporated into proteins or peptides, including GSH. Availability of transported cysteine or selenium can, therefore, affect the GSH levels and redox status of the epithelial cells themselves, as well as that of the adjacent cells located within the intestinal wall, including immune cells. 

Among amino acid transporters in GI epithelial cells, EAAT3 (excitatory amino acid transporter 3, EAAC1) is selective for cysteine and was initially cloned from GI epithelial cells [43]. Subsequent studies revealed that EAAT3 is most prominently expressed in the small intestine, especially in the terminal ileum [43, 44], a prominent site of inflammation in subjects with autism [45]. The highest levels were found in crypt cells and lower villus regions, the locus of multipotent stem cells that sustain the epithelial lining of the gut [44]. EAAT3 is a member of a family of Na^+^- and H^+^-dependent amino acid transporters named for their ability to transport glutamate and aspartate, but EAAT3 is unique in its preference for cysteine. EAAT3 exists in equilibrium between the endoplasmic reticulum and the plasma membrane, and its transport activity is regulated by the PI3 kinase signaling pathway, similar to insulin regulation of glucose transporters [46]. In resting cells, about 75% of EAAT3 resides inside the cell, where it is inactive, but activation of either PI3 kinase or protein kinase C increases the proportion of active transporters in the plasma membrane. Oxidative stress inhibits EAAT3 transport activity [47], and several cysteine residues have been identified as being critical for this mode of regulation, raising the possibility that they might be targets for glutathionylation. Oxidative stress affecting gut epithelium would, therefore, decrease EAAT3-dependent absorption of cysteine, with the local and systemic consequences of lower GSH levels.

Cells express a multitude of adaptive mechanisms that allow them to maintain homeostatic redox equilibrium, of which the Keap1/Nrf2 system is a prominent example. Nrf2 (nuclear factor erythroid 2-like 2) is a transcription factor protein with a high turnover rate, that is, tightly bound to Keap-1 (Kelch-like ECH-associated protein 1); however, in response to a more oxidative redox state, Nrf2 is released from Keap-1 and moves to the nucleus where it promotes expression of a large number of genes via binding to antioxidant response element (ARE) sites [48]. It has been recently demonstrated that Keap1/Nrf2 mediates up-regulation of EAAT3 in response to oxidative stress [49].

Similar to autism, GI disorders such as celiac disease and inflammatory bowel disease have an autoimmune component [50], and diets excluding gluten and/or casein (i.e., GF/CF diet) are frequently beneficial in both conditions. Prompted by the recognition that digestion of both gluten and casein yields peptides with opiate activity [51, 52], we recently examined the activity of these peptides on EAAT3-mediated cysteine uptake and redox status in human GI epithelial and neuronal cells and found that they significantly inhibited uptake [53]. Their action was associated with a decrease in GSH levels and the SAM/SAH ratio, along with alteration in the expression of redox-related genes. Important differences, however, were observed between peptides derived from bovine versus human casein, which may contribute to the well-recognized benefits of breastfeeding. 

### 3.3. Postnatal Epigenetic Regulation (PEP)

Methylation of DNA and histones is fundamental to epigenetic regulation of gene expression, providing factors affecting methylation capacity with an opportunity to affect development. S-adenosylmethionine (SAM) is the donor of methyl groups to more than 200 methyltransferase reactions, including DNA and histone methyltransferases [54], whereas its de-methylated form, S-adenosylhomocysteine (SAH), inhibits methylation by virtue of its competition with SAM. The SAM to SAH ratio determines methylation capacity for all methylation reactions, providing an exceptionally broad influence over cellular metabolism. 

**Figure 2:**
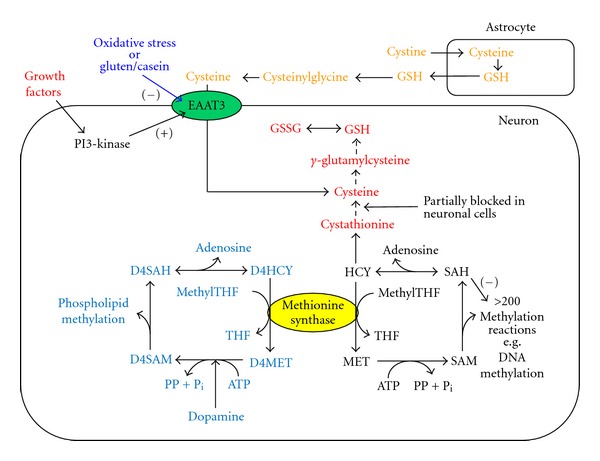
Redox and methylation pathways in neurons. The amino acid cysteine is rate-limiting for glutathione (GSH) synthesis and it is provided either by uptake from astrocyte-derived cysteine or by transsulfuration of homocysteine (HCY). The methionine cycle of methylation (lower right) depends upon both dietary methionine (MET) and remethylation of HCY by methionine synthase. Since formation of HCY from S-adenosylhomocysteine (SAH) is reversible and SAH inhibits methylation, decreased methionine synthase activity (e.g., caused by oxidative stress) both augments GSH synthesis and inhibits methylation reactions. Thus, redox status and methylation activity are closely linked. The D4 dopamine receptor carries out a cycle of dopamine-stimulated phospholipid methylation which is completely dependent upon methionine synthase and is therefore also sensitive to oxidative stress. Growth factors promote EAAT3 activity by increasing its location on the cell surface. EAAT3 activity is inhibited by oxidative stress and by food-derived opiate peptides.

The SAM/SAH ratio is responsive to the activity of methionine synthase (MS), the folate, and vitamin B12-dependent enzyme that converts homocysteine (HCY) to methionine (MET) as part of the methionine cycle of methylation (Figure 2). Its vitamin B12 (cobalamin) cofactor renders MS activity highly sensitive to oxidative stress; changes in cellular redox status (shifts toward reducing or oxidizing conditions) are able to exert epigenetic effects via their influence on MS and the SAM/SAH ratio. Recognizing this metabolic relationship, we previously proposed a “redox/methylation hypothesis” of autism, linking this neurodevelopmental disorder to oxidative stress induced by xenobiotic exposures [55], and now we apply this perspective to epigenetic programming of GI and immune function. 

During oxidative stress associated with GI inflammation, impaired EAAT3 activity will decrease availability of cysteine for GSH synthesis, leading to increased ROS levels, diminished MS activity, a decrease in the SAM/SAH ratio and a global decrease in methylation capacity, altering the pattern of DNA and histone methylation in epithelial cells. The distribution of EAAT3 suggests that this inflammation-induced epigenetic response will be most prominent in crypt stem cells of the terminal ileum, where it can influence both the proliferation and the functionality of the GI epithelium. Notably, the terminal ileum is also a critical location for folate and vitamin B12 absorption [56, 57]. 

Postnatal infant nutrition is most commonly provided by either human breast milk or bovine milk. Human breast milk is comparatively rich in sulfur-containing amino acids, and colostrum contains 2.5-fold higher levels versus mature milk [58]. A comparison of plasma cysteine levels in 12-week-old infants found significantly higher levels for breast-fed versus formula-fed infants [59]. Thus, breast feeding appears to provide a superior supply of the essential raw materials for synthesis of the antioxidant GSH. As this source of cysteine is made available, EAAT3 and the factors that modulate its activity serve as critical regulators of the extent to which cysteine may be absorbed. 

Breast milk contains a number of different growth factors, including insulin-like growth factor-1 (IGF-1), epidermal growth factor (EGF), vascular endothelial growth factor (VEGF), hepatocyte growth factor (HGF), and transforming growth factor-*β*1 and *β*2 (TGF-*β*1/*β*2), among others [60, 61]. These factors activate PI3 kinase and mitogen-activated protein (MAP) kinase signaling pathways to achieve their growth-promoting effects, which are associated with increased metabolic activity and its attendant increase in ROS production. We recently demonstrated the ability of IGF-1 and other PI3 kinase-activating growth factors to stimulate EAAT3-mediated cysteine uptake in cultured human neuronal cells, accompanied by an increase in GSH levels, an increased SAM/SAH ratio, and increased DNA methylation [62]. HGF, acting via the MAP kinase pathway, increases expression of the two enzymes which convert cysteine to GSH [63]. Thus, growth factors promote GSH synthesis via both increased cysteine uptake and transcriptional effects, corresponding to short-term and longer-term effects, respectively. These observations imply that breast-milk-derived growth factors can stimulate cysteine uptake and increase GSH in the GI epithelium, coincident with the initiation of postnatal digestion. The extent to which antioxidant resources (i.e., cysteine and GSH) are made available to the rest of the body depends upon the efficiency of this process. 

Human breast milk is also rich in selenium, and breast-fed infants have higher selenium levels than formula-fed infants [64]. A significant proportion (up to 30%) of breast milk selenium is in the form of glutathione peroxidase (GPx), which is hydrolyzed to selenocysteine during digestion. The importance of GPx activity in breast milk may be to combat oxidation, especially of vulnerable omega-3 fatty acids. However, GPx appears to also represent an important reservoir for delivery of selenium to the developing infant. As a structural analog of cysteine, breast-milk-derived selenocysteine can also be transported by EAAT3 and contributes to the antioxidant capacity and redox status [65].

As MS activity and the SAM/SAH ratio are responsive to redox status, it is reasonable to propose that the postnatal transition to independent nutrition is associated with epigenetic responses and that growth factor stimulation of cysteine uptake is a key event in this transition, which we call postnatal epigenetic programming or PEP. Although PEP is an ongoing body-wide process affecting the development of all tissues, but we specifically propose that the GI tract plays a uniquely important role as the source of nutritional support for metabolism, and that availability of antioxidant resources is the critical determinant of the level of metabolic activity that may be achieved without cellular damage. Epigenetic regulation of gene expression provides a molecular mechanism by which the functional state of the GI tract can be matched to the ongoing level of metabolic activity. In other words, the supply of antioxidant must be matched to the demand for antioxidant, and the rate of postnatal growth and development must be restricted so as to not exceed the supply provided by the GI tract.

As a developmental disorder in which the availability of GSH is reported to be reduced by almost 40%, autism can be viewed as a syndrome resulting from an imbalance between antioxidant supply and demand—essentially, a state of oxidative stress that interrupts the normal epigenetically based program of development. In the absence of a large-scale prospective study, it is not possible to determine whether the autism-associated GSH deficit is present from birth or begin during postnatal development. The former case would likely reflect a significant maternal deficit in antioxidant resources, and the finding that plasma levels of GSH and the SAM/SAH ratio are significantly decreased in mothers of autistic children [36] is consistent with such a possibility. Under the latter scenario, the imbalance might develop in a progressive manner during postnatal development if escalating metabolic activity and ROS production outstripped the ability of the GI tract to provide sufficient antioxidant, and inhibition of EAAT3-mediated cysteine uptake in response to developing oxidative stress would also exacerbate this self-reinforcing cycle. Postnatal exposure to agents which interfere with antioxidant capacity, including mercury, lead, pesticides, and other xenobiotics, could shift the redox balance sufficiently to initiate developmental regression secondary to impaired methylation and its epigenetic consequences. 

Microbes within the GI tract compete for nutrient resources in a normally symbiotic manner, but dysbiosis can intervene when the normal array of organisms is displaced or skewed. GI dysfunction, which is common in autism [8–12], may be associated with a failure of the GI epithelium to absorb antioxidant nutrients, such as cysteine or selenocysteine. Increased availability of these critical antioxidant nutrients for intestinal microbes may encourage the growth of previously restricted microorganisms, while diminishing the growth of others. Once established, the dysbiotic microbial population will decrease availability of antioxidant nutrients for absorption by further competing with the host, perpetuating the antioxidant deficit. 

The importance of PEP varies by cell and tissue types, depending upon the extent of the developmental maturation of specific cells and organs at birth. Delaying the development of selected neural networks (e.g., cortex and hippocampus) until after birth allows their programming to be guided by experience, in contrast to other networks (e.g., brainstem respiratory centers) which must function immediately upon birth. Indeed, unique features of human brain allow PEP to be dynamically exploited throughout the lifespan, providing the capacity for memory [66]. In a similar manner, maturation of the immune system is restrained until birth so as to optimize its response to environmental exposures, while at the same time minimizing its potential for autoimmunity. While this perspective encompasses the triad of neuronal, immune, and GI symptom domains in autism, the prominence of each component may vary among individuals, reflecting, in part, genetic differences in the vulnerability to oxidative stress and impaired methylation.

### 3.4. Redox Signaling in Human Brain

As previously noted, cysteine availability is limited for GSH synthesis. However, the level of cysteine in cerebrospinal fluid (CSF) is only one tenth the level in blood [67], meaning that the raw material for making antioxidants is much scarcer in the brain. Indeed, the GSH level in neurons is the lowest reported for any cell type [25], despite the fact that the brain utilizes oxygen at a ten times higher rate than other tissues. As illustrated in Figure 2, neurons obtain their cysteine from neighboring astrocytes, which release GSH that is subsequently broken down to cysteine, which is then available for uptake by neurons, depending upon the level of EAAT3 activity, which is controlled by growth factors. Growth factors utilize redox status to regulate neuronal function via the intermediate involvement of MS, a form of “redox signaling.” By allowing more cysteine into cells and increasing GSH synthesis, growth factors increase MS activity and increase DNA methylation. These increases in MS activity and DNA methylation lead to epigenetic changes in gene expression as well as many other metabolic effects through >200 methylation reactions, all affected by the SAM/SAH ratio. The increase in antioxidant level also allows cells to safely increase their oxidative metabolism (that is, their mitochondrial activity); the resultant increase in ATP supports a higher level of neuronal activity. In this way, redox signaling not only regulates gene expression but also controls the level of neuronal function.

A second brain-specific feature that augments redox signaling is a brain-specific limitation in the level of transsulfuration activity, which restricts conversion of HCY to cysteine [68]. This unique feature contributes to the low GSH level in neurons, making them more responsive to growth factor-induced EAAT3 activation. Together with the limited availability of extracellular cysteine, this makes epigenetic regulation exceptionally dynamic. Along with other methylation-dependent consequences of redox signaling, the long-term nature of epigenetic effects endows neurons with the ability to create “metabolic memories” that are coordinated with experience via the input of sensory systems. This view is consistent with recent studies identifying the central role of epigenetic changes in learning and memory formation [66, 69]. Compromised function of these redox-dependent systems could contribute to the types of cognitive function deficits and neurodevelopmental delays that are primary to autism. 

The restricted availability of GSH in the brain is partly compensated for by the presence of alternate mechanisms for sustaining antioxidant capacity, including an increased dependence upon selenoproteins. The brain has developed the capacity to retain selenium under conditions of scarcity, even when other tissues become depleted. Nonetheless, reliance on and retention of selenoproteins are an insufficient means of maintaining antioxidant defenses under certain conditions, such as exposure to mercury. Selenoproteins are exquisitely sensitive to inhibition by mercury, and any mercury that penetrates the brain will interfere with redox signaling and disrupt normal epigenetic regulation [70, 71]. Very tight binding of mercury by selenoproteins, especially to selenoprotein P, contributes to the long-term retention of mercury in the brain and its accumulation across the lifespan, and it has been proposed that selenoprotein P, which contains ten selenocysteine residues, has evolved to serve a specific role to protect against mercury toxicity [72]. 

In addition to conversion of HCY to MET, MS carries out a second reaction, providing methyl groups to the D4 dopamine receptor; the D4 receptor, when stimulated by dopamine, subsequently transfers these methyl groups to membrane phospholipids—a unique activity carried out by the D4 dopamine receptor [73]. Genetic variants of the D4 receptor (such as the 7-repeat variant) have been linked to novelty-seeking behavior and are important risk factors for attention-deficit/hyperactivity disorder (ADHD) [74]. Although the role of dopamine-stimulated phospholipid methylation remains incompletely understood, we first proposed that it facilitates attention by modulating the frequency of neural networks, promoting their synchronization [75]; others subsequently confirmed dopamine-stimulated phospholipid methylation to be critically involved in neuronal synchronization during attention [76]. As D4 receptor-mediated phospholipid methylation is absolutely dependent upon MS activity, it is thus also dependent on EAAT3, raising the possibility that ADHD might involve a decrease in MS activity secondary to oxidative stress. 

### 3.5. Redox and the Immune Response

In the past several years, a previously unappreciated role for redox regulation of the immune response has been elucidated, involving extrusion of GSH from activated antigen presenting cells (APCs, e.g., dendritic cells, macrophages, or B cells), followed by release of its cysteine, which is then taken up by T cells [77] This relationship is analogous to the relationship between astrocytes and neurons illustrated in Figure 2. Uptake of this cysteine by naïve CD4^+^ CD25^−^ effector T cells (Teff) leads to their activation and proliferation, in association with increased GSH synthesis and the epigenetic consequences thereof. Activated Teff cells release cytokines that attract other cells and promote inflammation, as well as stimulate B cell activation and antibody production (Helper T cells). However, the extent of Teff cell activation is limited by CD4^+^ CD25^+^ Foxp3^+^ regulatory T cells (Treg) that compete with Teff cells for available extracellular cysteine, thereby suppressing immune and autoimmune responses [77]. 

**Figure 3:**
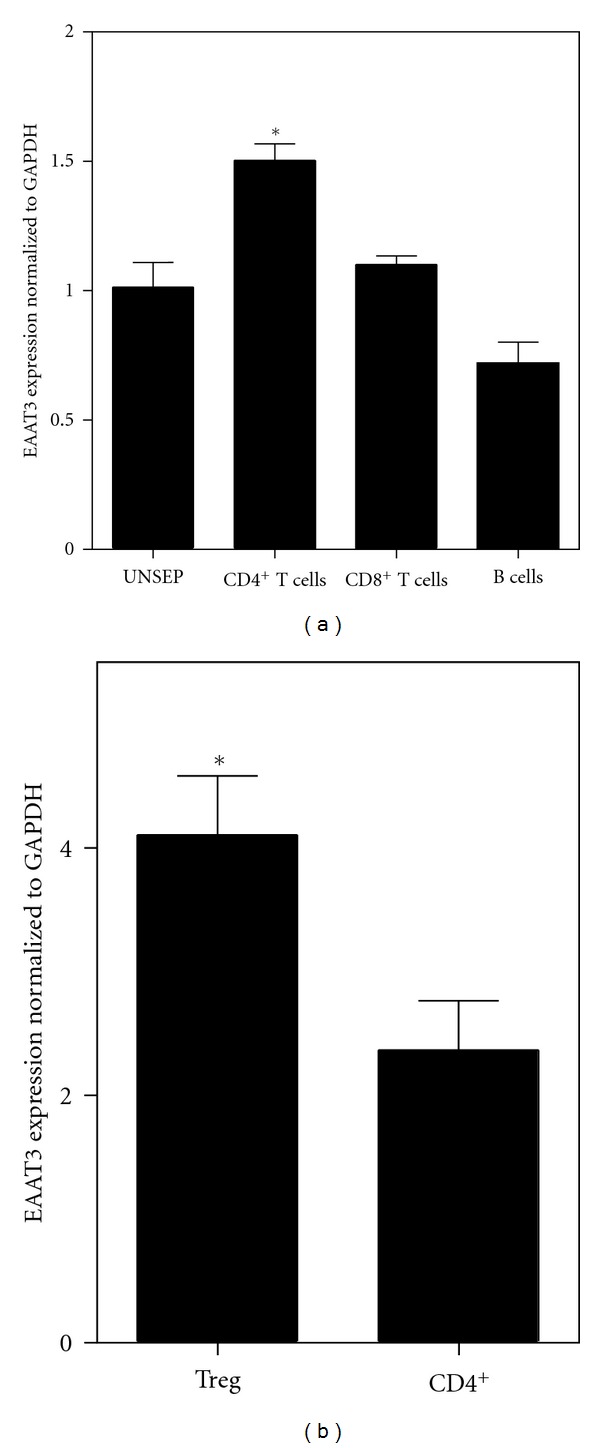
Expression of EAAT3 in murine lymphocyte subsets. Spleen and lymph node-derived lymphocytes were separated by magnetic cell sorting, followed by qRT-PCR analysis for EAAT3 (EAAC1) expression. (a) EAAT3 expression was significantly higher (*P* < 0.05) in CD4^+^ T cells as compared to unseparated lymphocytes (UNSEP), CD8^+^ T cells, or B cells. (b) EAAT3 expression was significantly higher (*P* < 0.05) in FoxP3^+^ Treg cells.

Recognizing this newly described mode of T cell regulation, we hypothesized that EAAT3 might be involved in cysteine uptake. To evaluate this possibility, we used qRT-PCR to determine whether EAAT3 was expressed in murine T cells and compared the level of expression in T cell subsets, separated on the basis of surface epitopes by magnetic cell sorting. As illustrated in Figure 3(a), EAAT3 expression was detected in unseparated cells, as well as in CD4^+^ and CD8^+^ T cells and B cell populations, with the highest expression level in CD4^+^ cells. Further analysis showed that the expression of EAAT3 by Treg cells is almost two-fold higher than that in unseparated CD4^+^ cells (Figure 3(b)). Thus, EAAT3 expression by immune cells provides a mechanism for them to take up cysteine in a competitive manner, and higher EAAT3 expression in Treg cells may underlie the ability of this cell subset to limit the immune response and restrict autoimmunity.

**Figure 4:**
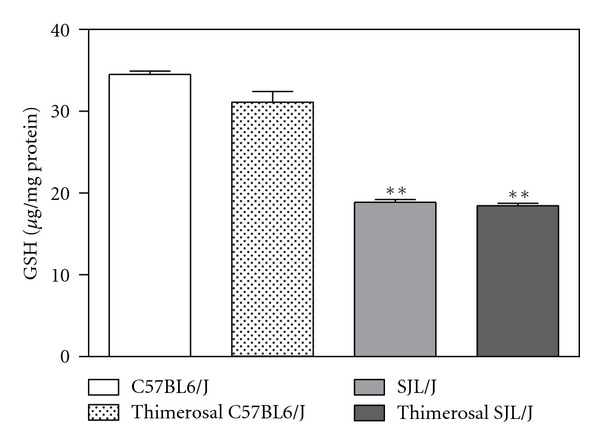
GSH levels in frontal cortex of SJL/J and C57BL6/J mice with or without thimerosal treatment. GSH levels were significantly lower (*P* < 0.05) in cortex of SJL/J autoimmune-prone mice compared to C57BL6/J strain mice. GSH levels were not affected by thimerosal treatment.

**Figure 5:**
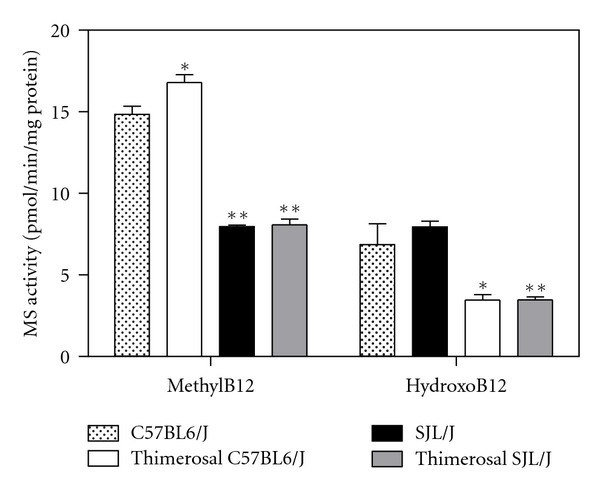
Methionine synthase activity in cortex of SJl/J and C57BL6/J mice with or without thimerosal treatment. Methionine synthase activity was measured in the presence of either methylcobalamin (MethylB12) or hydroxocobalamin (HydroxoB12). Activity was significantly lower in SJL/J autoimmune-prone mice compared to the C57BL6/J strain, irrespective of thimerosal treatment. Activity was higher in the presence of methylcobalamin. *P < 0.02 compared to C57BL6/J group, **P < 0.001 compared to C57BL6/J group.

**Figure 6:**
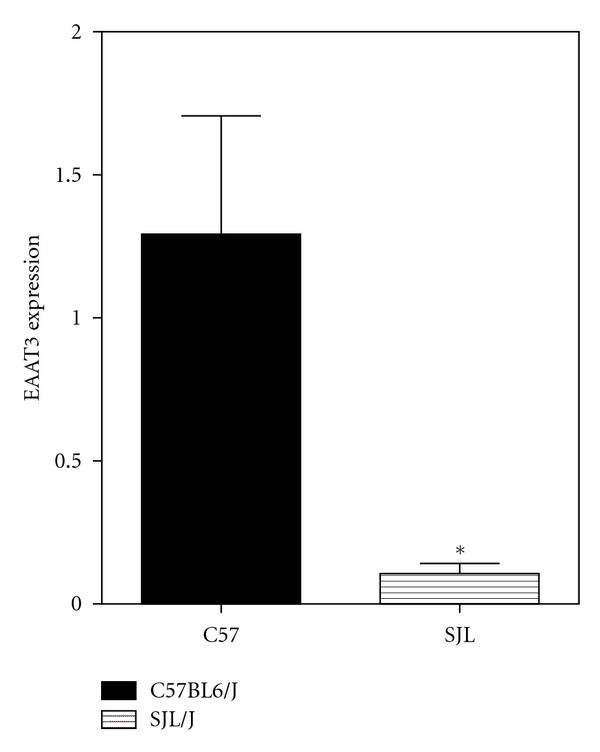
Expression of EAAT3 in CD4^+^ T-cells from C57BL6/J and SJL/J mice. Spleen and lymph node-derived lymphocytes were separated by FACS analysis cell sorting, followed by qRT-PCR analysis for EAAT3 (EAAC1) expression. EAAT3 expression was significantly higher in CD4^+^ T-cells from C57BL6/J mice versus autoimmune-prone SJL/J mice (*P* < 0.05).

 The SJL/J strain of mice has been widely employed as a model for autoimmunity, but these mice also exhibit deficits in GSH, similar to those reported in autistic children. In SJL/J mice, the deficit is due to mutations in the enzymes which synthesize GSH; factors underlying GSH deficits in autism are as yet unknown. In a previous study, we showed that the thiol-reactive compound thimerosal caused growth impairment, as well as neurodevelopmental and neurochemical effects in SJL/J mice, but not in C57BL6/J mice, including upregulated brain levels of EAAT3 [21]. To further characterize the role of redox and methylation status in autoimmunity, we measured both GSH levels and MS activity in the liver and brain of control and thimerosal-treated SJL/J mice, as compared to C57BL6/J mice. GSH levels were significantly lower in both liver and brain of SJL/J as compared with the levels found in C57BL6/J mice, but they did not differ as a function of thimerosal treatment in either strain (Figure 4). Methionine synthase activity was also significantly lower in SJL/J versus C57BL6/J mice, but not different in thimerosal-treated animals (Figure 5). These results confirm the occurrence of low GSH in the autoimmune-prone SJL/J strain, along with the novel finding that MS activity is reduced, although neonatal exposure to thimerosal did not alter these features. Expression of EAAT3 was significantly lower in CD4^+^ T-cells from SJL/J mice versus C57BL6/J mice, suggesting that autoimmunity is associated with impaired capacity for cysteine uptake (Figure 6). Together these findings provide support for the roles of oxidative stress and impaired methylation in autoimmunity.

**Figure 7:**
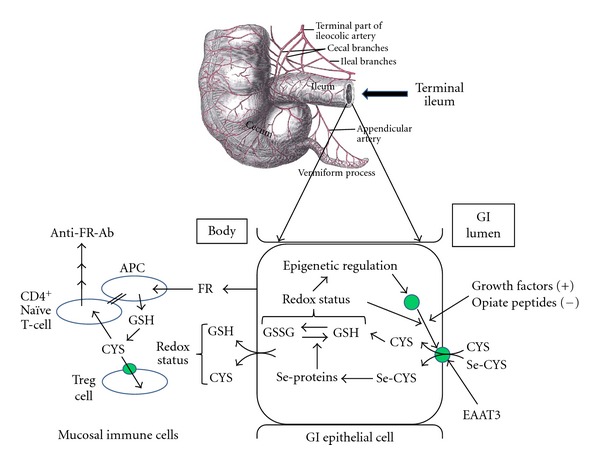
Cysteine and selenocysteine uptake in the terminal ileum regulates autoimmunity. EAAT3 transports diet-derived cysteine (CYS) and selenocysteine (Sel-CYS) into GI epithelial cells in the terminal ileum. Within the cell, cysteine is directed to GSH synthesis and selenocysteine is incorporated into selenoproteins, which maintain GSH in its reduced state. EAAT3 activity is regulated by multiple factors, including intracellular redox status, Nrf2-dependent transcription, growth-factor-dependent translocation to the cell surface, and the inhibitory effects of casein and gluten-derived opiate peptides. Systemic availability of cysteine and GSH depends upon these terminal ileum events, as does the local mucosal environment. Immune cells in the terminal ileum can be a source of auto-antibodies, including anti-folate receptor (FR) antibodies, which are commonly present in subjects with autism [80]. Under oxidative stress conditions, EAAT3-mediated uptake of cysteine by Treg cells is impaired, limiting their suppression of naïve CD4^+^ T cells, and increasing autoimmune responses to antigens present locally, such as the folate receptor.

Abnormal immune function, including, but not limited to, increased autoimmunity, has been extensively documented in autism. Of particular relevance are autoantibodies targeting brain and neuronal proteins [2, 3, 5] as well as autoantibodies targeting the folate receptor [78–80]. The latter were initially linked to the relatively rare cerebral folate deficiency syndrome [78] but recently were found to be present in a high proportion (75%) of autistic subjects [80], and a casein-free diet greatly reduced the circulating level of anti-folate receptor antibodies [79]. The number of circulating Treg cells was reported to be significantly lower in autistic subjects, with 73% showing abnormally low levels [4]. The Treg cell decrease was more prominent in the more severe cases, as well as among individuals with allergic manifestations such as asthma and atopic dermatitis. Although autistic subjects with low Treg cells did not manifest clinical or laboratory-based signs of autoimmunity (arthritis or arthralgia, elevated erythrocyte sedimentation rate, or leucopenia), the frequency of autoimmunity was higher in their families. The same researchers found elevated markers of oxidative stress (increased plasma F2-isoprostane and/or decreased GPx activity) in 89% of autistic subjects and evidence of antineuronal antibodies in 55%, leading them to suggest a link between oxidative stress and autoimmunity [5]. Notably, expression of Foxp3, the transcription factor which defines Treg cells, is epigenetically regulated, with increased expression when DNA methylation is decreased [81], and Treg cell levels are elevated in male, but not female, SJL mice [82]. As illustrated in Figure 7, these observations suggest that autoimmunity in autism may be related to inflammation in the distal ileum, where impaired activity of EAAT3 in Treg cells can lead to formation of autoantibodies directed against the folate receptor. 

## 4. Conclusions 

The prominence of neurological, GI, and immune symptoms in autism suggests a shared mechanism of dysregulation that may relate to the significant deficits in systemic reservoirs of antioxidant GSH reported in this neurodevelopmental disorder. Impaired GI absorption of cysteine, the essential GSH precursor, is proposed as a crucial factor in the pathogenesis of this triad of symptoms, wherein reduced cysteine availability leads to a condition of local and systemic oxidative stress, and subsequent disruption of normal epigenetic regulation of gene expression. In some cases, the extent of impaired cysteine absorption may be severe enough to result in overt GI inflammation, while in other cases the restriction may only alter immune and/or neurological development and function. Awareness of the redox-based linkage between GI, brain, and immune systems serves to illuminate a number of diseases whose origins may reflect the critically important roles of PrEP and PEP. Indeed, adaptive epigenetic responses to changes in redox status are likely to play a critical role in diseases arising across the lifespan, especially those that can be traced to environmental exposures that interfere with antioxidant homeostasis.
